# Adult Ocular Toxocariasis Mimicking Ciliary Body Malignancy

**DOI:** 10.1155/2014/368907

**Published:** 2014-10-09

**Authors:** Ahmad M. Mansour, Bachir Abiad, Fouad I. Boulos, Ramzi Alameddine, Fadi C. Maalouf, Alaa Bu Ghannam, Rola N. Hamam

**Affiliations:** ^1^Department of Ophthalmology, Retina Service, American University of Beirut, P.O. Box 113-6044, Beirut, Lebanon; ^2^Department of Ophthalmology, American University of Beirut, P.O. Box 113-6044, Beirut, Lebanon; ^3^Department of Pathology and Laboratory Medicine, American University of Beirut Medical Center, P.O. Box 11-0236, Riad El Solh, Beirut 110 72020, Lebanon; ^4^Uveitis Division, Department of Ophthalmology, American University of Beirut, P.O. Box 113-6044, Beirut, Lebanon

## Abstract

*Purpose*. To discuss an unusual presentation of ocular toxocariasis. *Methods*. Case report. *Results*. A 40-year-old woman presented with decreased vision in the left eye with a long history of recurrent red eye from uveitis. Eosinophilia and positive ELISA titers for *Toxocara canis* favored the diagnosis of ocular toxocariasis. Over 3 months, an anterior scleral mass had a rapid growth raising the possibility of medulloepithelioma, which rarely can mimic uveitic syndromes. Surgical plan changed from local excision to enucleation. Histopathology demonstrated a large homogeneous mass of chronic inflammatory cells with inflammation of the overlying thinned out sclera, medial rectus insertion, and limbal cornea. The triad of peripheral granuloma, eosinophilia, and positive blood serology established the diagnosis of ocular toxocariasis. *Conclusions*. Ocular toxocariasis can mimic ocular malignancy such as medulloepithelioma in adults and rarely presents as an anterior scleral mass.

## 1. Introduction

Ocular toxocariasis (OT) is caused by ocular infestation by larvae of the roundworm* Toxocara* species typically manifesting in older children with history of soil ingestion or exposure to dogs [1, 2]. OT presents clinically as a peripheral retinal inflammatory mass with vitritis simulating rarely an endophytic retinoblastoma [3]. The second manifestation is a posterior pole subretinal granuloma with minimal vitritis and rarely resembling exophytic retinoblastoma [1]. Cataract formation in OT [4] consists of small, round, white lens opacities resembling retinal granulomas located primarily in the lens midperiphery and in the subcapsular level. OT was thought to be an uncommon cause of uveitis in adults [1, 2, 5]; however more recent data points to its higher prevalence in adults than originally thought [6]. We present the case of an adult who developed recurrent red eye since early childhood, recurrent uveitis, cataract, and anterior scleral mass mistaken for malignancy.

## 2. Case Report

**Figure 1:**
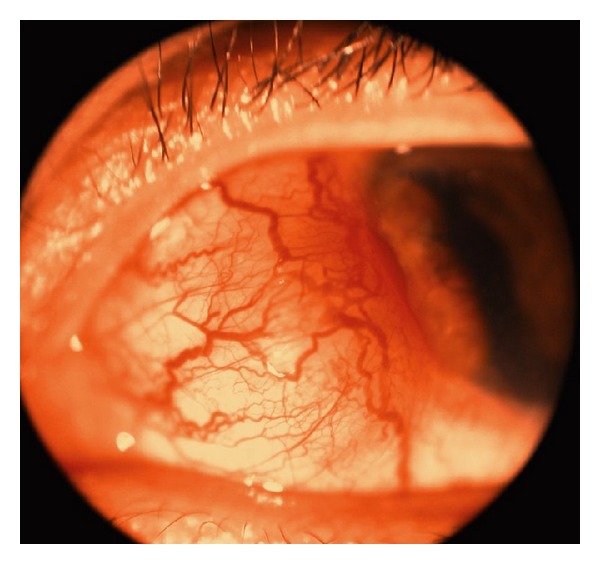
Slit lamp photograph of the left eye 1 month after initial presentation showing a nasal anterior scleral bulge reaching the limbus with prominent dilated episcleral vessels.

**Figure 2:**
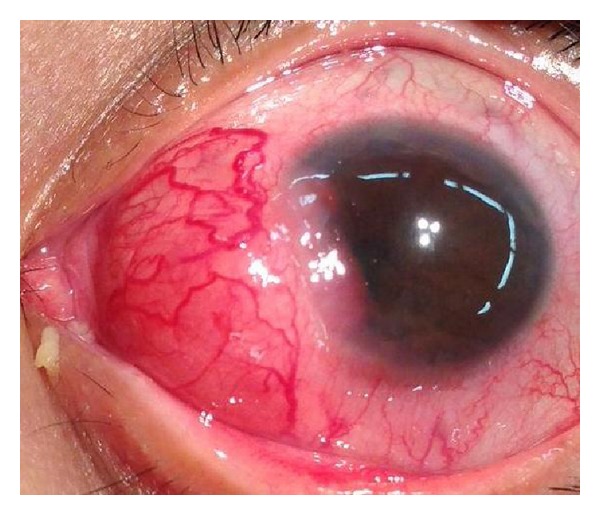
The anterior scleral mass of the left eye has grown in all dimensions and invaded the cornea. Photograph was taken in the operating room 3 months after Figure 1.

**Figure 3:**
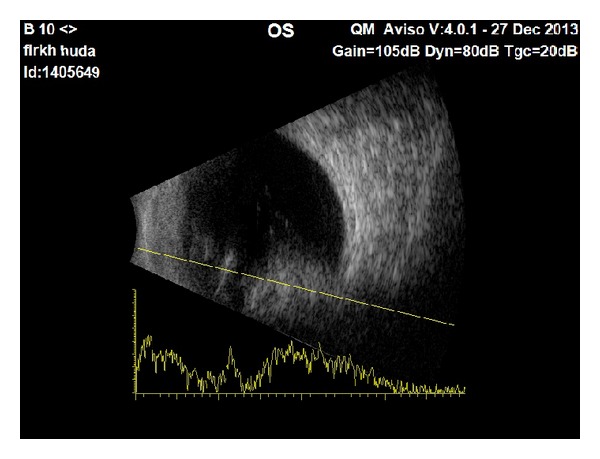
B-scan of the eye shows an intraocular homogeneous mass behind the posterior lens capsule and involving the retina and vitreous. The mass has medium to high reflectivity.

**Figure 4:**
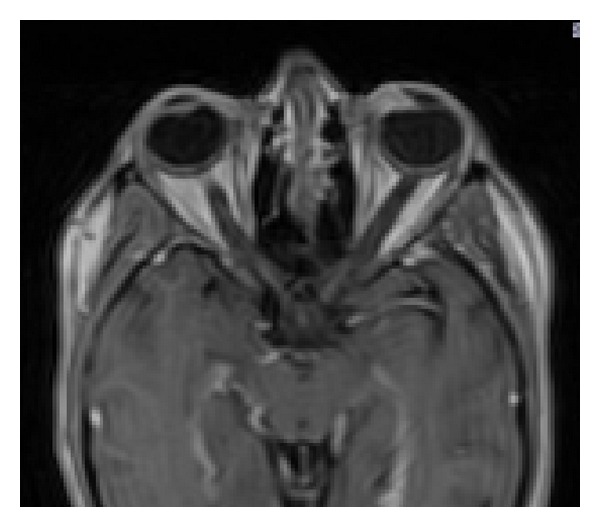
Magnetic resonance imaging scan demonstrates an anterior scleral mass behind the iris plane and with nasal scleral bulge.

This 40-year-old woman presented for recurrent redness of the left eye since the age of 6 years. She was diagnosed to have unilateral uveitis at the age of 14. She underwent uneventful phacoemulsification with implant at the age of 38. Systemic diseases included idiopathic thrombocytopenia (controlled by splenectomy), factor V Leyden deficiency (maintained for life on acenocoumarol), and familial Mediterranean fever (controlled on colchicine). The patient had no encounter with pets but is a frequent eater of raw meat. Family history was negative for malignancy and was positive for psoriasis in a sister. Prior to her presentation, an outside ophthalmologist prescribed oral corticosteroids. The patient had to stop oral corticosteroids because of side effects and was maintained on topical corticosteroids. Visual acuity was finger counting near face in the left eye. Slit lamp examination revealed nasal scleral inflammation, nasal iridolenticular touch, mild iritis, 360° unbreakable posterior synechiae preventing view of the fundus, and nasally drawn miotic pupil. The initial impression was sclerouveitis. Laboratory investigations included blood count, antinuclear antibodies, anticardiolipin, Venereal Disease Research Laboratory, angiotensin converting enzyme, tests for* Toxocara canis*, purified protein derivative skin test, and chest radiograph. Positive results included eosinophilia of 18% (normal 0–5%) and positive tests for* Toxocara canis* (Bioscientia, Ingelheim, Germany). Both* Toxocara canis* IgG and* Toxocara canis* IgM yielded positive reaction with the specific antigens 24, 28, 30, and 35 kD using Western Blot. By enzyme-linked immunosorbent assay (ELISA),* Toxocara canis* IgG yielded high concentrations of 30.2 U/mL (negative < 8.5 U/mL). Our initial working diagnosis was ocular toxocariasis (OT) and the patient was advised a 30-day course of oral albendazole as well as local excision of the scleral mass (Figure 1). The scleral mass kept on growing in a rapid fashion while the patient did not take antihelminthic therapy and was hesitant for surgery. Three months after presentation, the bulge of the sclera was mimicking clinically a ciliary body tumor, namely, medulloepithelioma (Figure 2). The mass has extended to involve the cornea and retina. Vision then had dropped to hand motion. B-scan (Figure 3) revealed a large intraocular mass of medium to high reflectivity behind the posterior capsule extending to the peripheral retina. MR orbit (Figure 4) revealed 12 × 13 × 9 mm lesion arising from the medial aspect of the iris to the medial canthus which was isointense on T1 and hypointense on T2 along with intense enhancement after gadolinium injection suggestive of medulloepithelioma or melanoma. Although our initial diagnosis was probable ocular toxocariasis, this rapid growth and the technical difficulties of total resection of this huge mass raised the possibility of leaving residual tumor in case of malignancy. After extensive discussion with the patient and family, the decision for enucleation was taken. Patient consented for surgery.

**Figure 5:**
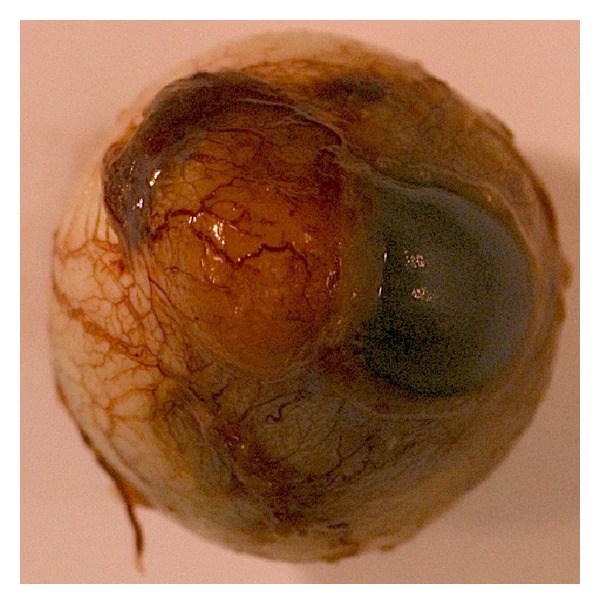
Gross view of the eyeball with nasal mass involving cornea and anterior sclera.

**Figure 6:**
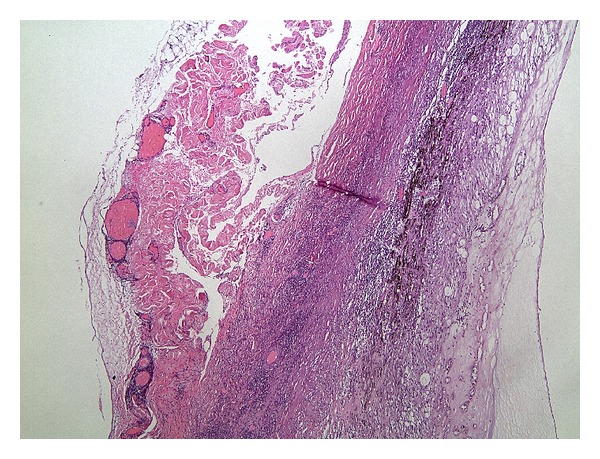
Inflammatory cell infiltration of the peripheral anterior vitreous base, sclera, and medial rectus insertion (hematoxylin and eosin stains).

**Figure 7:**
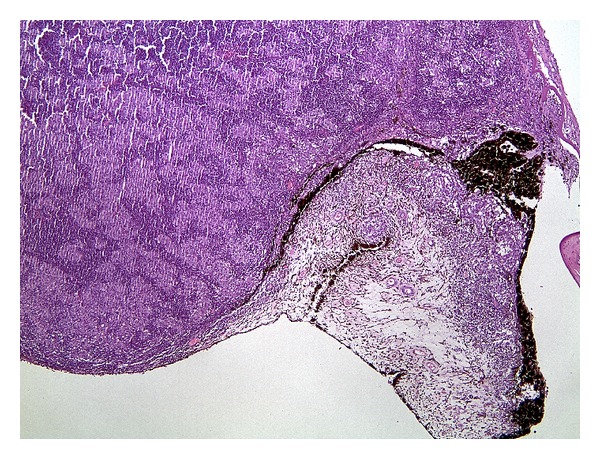
Homogeneous mass in the ciliary body area pressing on the posterior iris plane consisting of a dense population of lymphocytes with histiocytic aggregates (hematoxylin and eosin stains).

**Figure 8:**
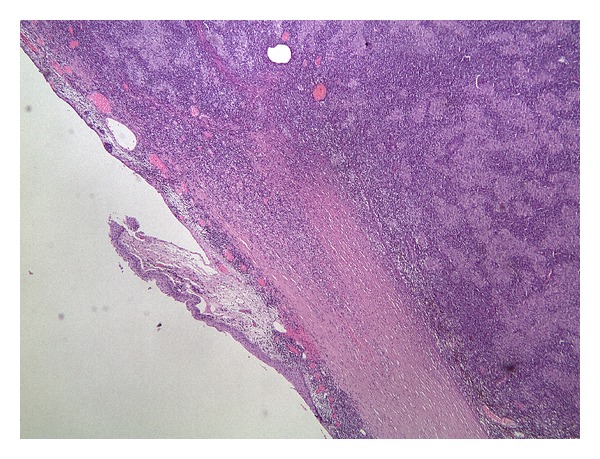
Nasal mass has invaded part of the limbal cornea and most of the sclera (extreme scleral thinning) (hematoxylin and eosin stains).

The globe was enucleated (Figure 5). The medial rectus muscle was lost during isolation of the muscle as the insertion was very friable overlying the scleral mass (Figure 6). The mass measured 13 mm by 13 mm by 5 mm (horizontal × vertical × height) (Figures 7 and 8). Upon sectioning the globe, the vitreous was severely liquefied and the posterior pole was grossly normal.

**Figure 9:**
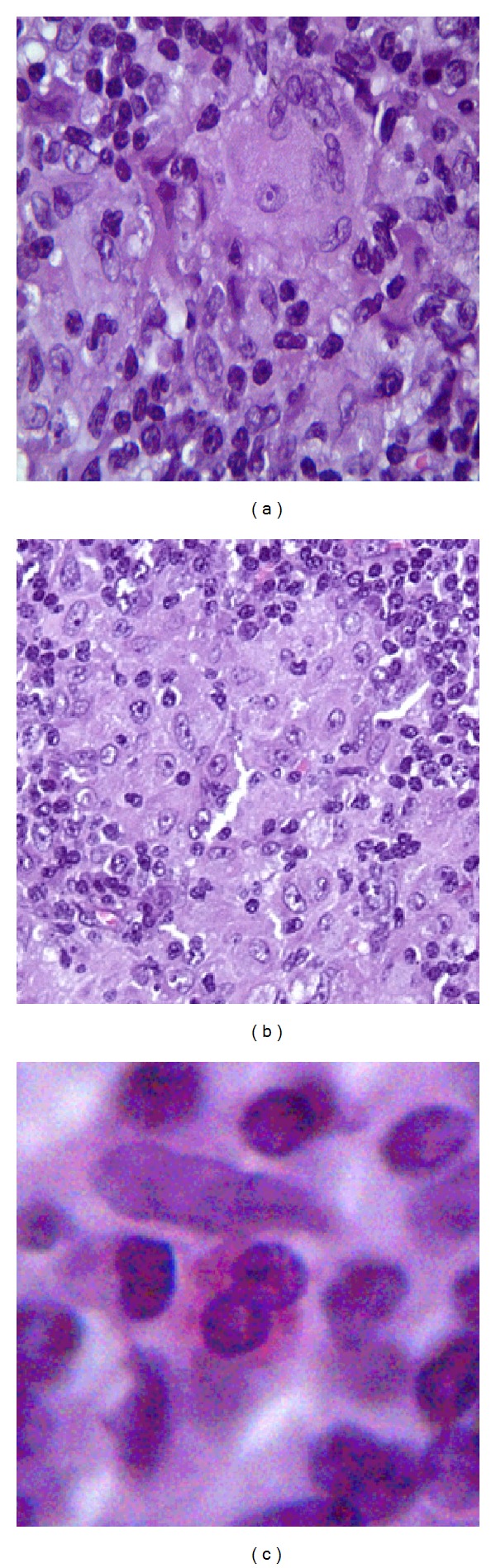
Giant cells (a), histiocytic aggregates (b), and eosinophils (c) are seen within the mass.

The whole mass consisted of lymphocytes, clusters of histiocytes, scattered giant cells, and few eosinophils (Figure 9). No larva or microorganisms could be detected on serial sectioning of the mass. Both Gomori methanamine Silver and acid fast bacillus stains were negative. Reactive germinal centers were focally present. Immunostains were performed on formalin-fixed paraffin-embedded sections using a polymer detection system with controls. There was a polyclonal population of CD3 and CD20 positive lymphocytes. The pathologic impression was granulomatous inflammation.

## 3. Discussion

OT is a zoonotic disease transmitted by ingestion of embryonated eggs from contaminated water, raw vegetables, or meat (chicken and lamb), or via geophagia. The parasite reaches the eye via the ciliary vessels to the choroid or via the central retinal arteries to the retina. OT is typically a monocular disease of young children [5], and its clinical findings include posterior and peripheral retinochoroiditis, optic papillitis, and endophthalmitis [7, 8]. The inflammatory response created by ocular involvement may result in epiretinal membrane formation, traction retinal detachment, and combined traction-rhegmatogenous retinal detachment [2]. The granuloma consists of lymphocytes, epithelioid cells, foreign body giant cells, and eosinophils.

OT remains a clinical diagnosis [2]. Ancillary tests include immunotesting and ultrasound biomicroscopy. Specific immunotesting is of importance but may be negative as* Toxocara* antigens may localize exclusively in vitreous humor [7]. Ultrasound biomicroscopy may be helpful with specific findings (pseudocystic transformation of the peripheral vitreous) in some eyes with a presumed diagnosis of peripheral toxocariasis [2].

The treatment consists of a 30-day course of the antihelminthic oral albendazole (10 mg/kg of body weight/day in two divided doses). An antihelminthic agent should be considered in the setting of an active systemic infection or if alive, motile larva is seen by ophthalmoscopy. In case of a large granuloma like our case, granuloma surgery (eyewall resection with scleral patch) appears to yield good clinical results and allows histological confirmation of the diagnosis. Vitrectomy is needed for epiretinal membranes, tractional or rhegmatogenous retinal detachment, or long standing vitreous debris [1, 2]. Cryotherapy can lead to resolution of peripheral granuloma especially if associated with a vasoproliferative mass [9, 10]. Corticosteroids are indicated to control uveitis attacks.

Eyes with OT are rarely enucleated for suspicion of retinoblastoma [3, 7, 8] in children. A second cause for enucleation of eyes with OT is for the presence of a secondary retinal vasoproliferative tumor [9, 10]. Similar to cases resembling retinoblastoma or vasoproliferative growths, the current case posed a problematic issue in diagnosis and management. The sudden growth of the tumor confused our preliminary diagnosis of OT and suggested the presence of a malignancy. Other differentials in our case include sarcoidosis [11], tuberculosis [12], colobomatous dysplasia of anterior uvea [13], and fungal and parasitic infections. Gopal et al. [12] described the successful eyewall excision in a case of tuberculous granuloma similar to the current case. Medulloepithelioma is occasionally a mimicker of uveitis in adults [14], but ultrasound may assist in the diagnosis as medulloepithelioma typically shows a mass with cystic spaces. Medulloepithelioma is the most common congenital tumor of the nonpigmented ciliary epithelium and can present in adulthood as a ciliary body mass, lens coloboma, lens subluxation, cataract, and cyclitic membrane [15–17]. The diagnosis is usually delayed for several years [14]. On gross examination, medulloepitheliomas appear as fleshy tan or white cystic masses in the ciliary body region with chalky gray-white deposits composed of hyaline cartilage. These tumors may extend anteriorly into the iris or posteriorly into the vitreous and retina. Moreover, such tumors demonstrate high internal reflectivity by B-scan while magnetic resonance imaging showed hyperintensity on T1-weighted scans and hypointensity on T2-weighted scans with homogeneous enhancement after gadolinium administration.

The diagnosis of OT is presumptive because a definite diagnosis requires actual demonstration of the larva in the patient eye. The triad of blood eosinophilia, positive blood titers for* Toxocara canis*, and histologic diagnosis of granuloma favor the clinical diagnosis of OT. Cataract surgery is known to increase inflammation in eyes with uveitis and could have exacerbated the inflammatory process in the present case. Also cataract surgery could have contributed to scleral thinning and scleral bulge as simple trauma to the sclera (grasping with forceps, cautery of episcleral vessels, and scleral wound) during surgery could trigger scleral necrosis [18]. Finally, scleritis could be related in part to the coexistence of familial Mediterranean fever as reported in the literature [19–21]. The absence of larva on serial cuts of the mass could be related to the gradual slow disintegration of the dead larva with recurrent bouts of inflammation since the age of 6.

## 4. Conclusions

The roundworm parasite in OT can cause visual impairment [5] (uveitis, cataract, retinal detachment, endophthalmitis, and submacular granuloma) and should be considered a possible causative agent of posterior or diffuse uveitis and considered in the differential diagnosis of retinoblastoma and retinal vasoproliferative tumor in children and medulloepithelioma in adults.
